# Distinct intracardiac electrogram waveforms with perforation during left bundle branch area pacing implantation

**DOI:** 10.1093/europace/euag010

**Published:** 2026-02-17

**Authors:** Heli Tolppanen, Valerian Valiton, Samuel Stempfel, Haran Burri

**Affiliations:** Cardiac Pacing Unit, Department of Cardiology, University Hospital of Geneva, rue Gabrielle Perret Gentil 4, Geneva 1211, Switzerland; Heart and Lung Center, Helsinki University Central Hospital, Helsinki, Finland; Cardiac Pacing Unit, Department of Cardiology, University Hospital of Geneva, rue Gabrielle Perret Gentil 4, Geneva 1211, Switzerland; Cardiac Pacing Unit, Department of Cardiology, University Hospital of Geneva, rue Gabrielle Perret Gentil 4, Geneva 1211, Switzerland; Cardiac Pacing Unit, Department of Cardiology, University Hospital of Geneva, rue Gabrielle Perret Gentil 4, Geneva 1211, Switzerland

**Keywords:** Left bundle branch area pacing, Conduction system pacing, Perforation, Complications, Electrogram, Current of injury

## Abstract

**Aims:**

Perforation during left bundle branch area pacing (LBBAP) results in a fall in the current of injury (COI) amplitude in the unipolar unfiltered electrogram (iEGM), but systematic waveform analyses have not been performed. Our aim was to investigate unipolar iEGM waveforms during perforation and to compare them to those at the final lead position.

**Methods and results:**

The iEGMS of consecutive patients who had perforation during LBBAP implantation were systematically analysed. A total of 92 patients with perforation were included. In the unfiltered channel, sensed COI amplitude was lower with perforation [3.0 (1.5–4.1) mV] than at the final lead position [14.0 (9.2–17.5) mV, *P* < 0.0001], as was also the case during pacing. Patients with narrow QRS/non-LBBB typically had wide negative (QS) waveforms during sensing (in 67% of cases), whereas those with LBBB/paced rhythm had positive (wide R/RS) morphologies (in 93% of cases). In the former subgroup, a sensed Q or S amplitude > COI amplitude (which is easy to eyeball during lead deployment) had a sensitivity of 86% and a specificity of 93% for diagnosing perforation. Waveforms during macroperforation (with loss of capture, *n* = 27) differed compared to microperforation (with preserved capture, *n* = 65), with significantly lower COI amplitudes, more frequent QS morphology, and rarer sharp multiphasic components in the ventriculogram of the filtered channel.

**Conclusion:**

Beyond COI amplitude, additional iEGM waveform parameters may be used to evaluate the presence of LBBAP perforation and should be carefully monitored during lead deployment to improve safety.

## Graphical Abstract

**Graphical Abstract:**
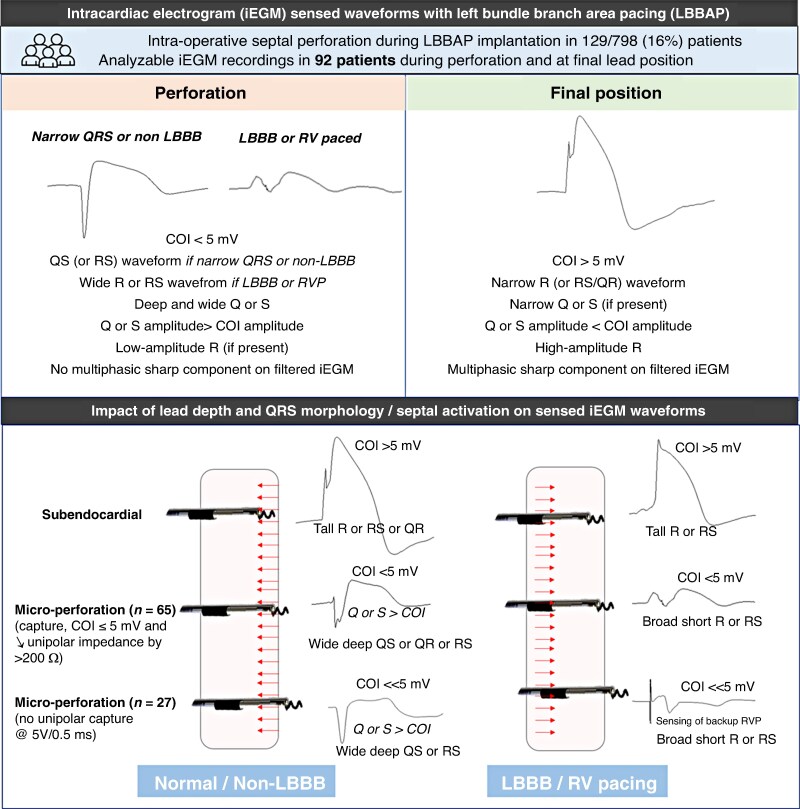
Waveforms during left bundle branch area pacing perforation and at final lead position. COI = current of injury; iEGM = intracardiac electrogram; LBBB = left bundle branch block; RVP = right ventricular pacing

What’s new?

- In addition to current of injury amplitude, intracardiac waveforms (Q, R, and S waves) are significantly different during perforation compared to the final lead position.
- Patients with left bundle branch block (LBBB) or right ventricular pacing (RVP) have a distinct broad R/RS sensed waveform during perforation compared to those with a narrow QRS/non-LBBB, who most often have a QS pattern.
- A Q or S amplitude > COI amplitude is frequently encountered during perforation in patients who have a narrow QRS or non-LBBB.
- A sharp multiphasic component of the ventriculogram in the filtered channel is less frequently observed during perforation as compared to the final position.
- Intracardiac waveforms during pacing may be useful to guide lead deployment. However, they may differ compared to during sensing, particularly with micro-perforation, so pacing should be periodically interrupted when paced current of injury approaches 10 mV.

## Introduction

Left bundle branch area pacing (LBBAP) is gaining momentum as a more physiological alternative to right ventricular (RV) pacing and biventricular pacing.^1–7^ Peroperative septal perforation during LBBAP implantation is relatively common, as achieving conduction system capture requires positioning the lead tip at close proximity to the left bundle trunk or to its fascicles, which lie in the left ventricular subendocardium. In recent clinical studies and registries, the incidence of peroperative perforation is up to 14%.^8–14^ There are no consequences if it is recognized promptly and the lead is repositioned.^12,14,15^

Complete or macroperforation is easy to recognize due to the complete loss of capture, sometimes with fluoroscopic visualization of free lead movement in the left ventricular cavity. Recognizing partial or microperforation, where only part of the screw has perforated, is, however, much more challenging, as thresholds may be preserved.^8,16,17^ Parameters that have been used to indicate lead depth and distance from the left ventricular subendocardium are the presence of a fascicular potential, pacing impedance, and unipolar sensed myocardial current of injury (COI) amplitude.^10,11,18^ Low-sensed COI amplitude(<5 mV) has been associated with perforations during LBBAP implantation in several reports, both in human^10,11,18^ as well as in animal studies,^17,19^ and it is advised to carefully monitor this parameter during lead deployment.^8,16^ This phenomenon can be explained by the drop in myocardial mass surrounding the lead tip (which generates the COI). However, COI amplitude is significantly affected by high-pass filters, the settings of which may differ according to physician preference or electrophysiological recording systems and pacing sense analysers (PSAs).^20^ In addition, COI may potentially be impacted by the septal substrate (e.g. fibrosis or amyloidosis), where low values may not necessarily reflect perforation. Another parameter is pacing impedance, which has been shown to drop to <450 Ω with perforated lumenless leads.^11^ However, impedance values depend upon lead characteristics (e.g. length) as well as the laboratory setup (connector cables, etc.). A drop of >200 Ω with respect to the maximum value has been proposed to monitor perforation,^21^ but this parameter has not been systematically studied. For these reasons, it is desirable to explore additional parameters to evaluate perforation.

There have been few studies, which have specifically studied intracardiac electrogram (iEGM) waveforms during LBBAP perforation, with at most 30 cases, and without detailed measurements of waveform amplitude and duration^11,17,19^

Our aim was therefore to systematically evaluate iEGM waveforms and COI during perforation and at the final lead position in patients undergoing LBBAP implantation.

## Methods

Consecutive patients who underwent LBBAP implantation at the University Hospital of Geneva were recruited. All patients were part of the Geneva Conduction System Pacing registry, which has been approved by the institutional ethics committee, and had given written informed consent. The study was conducted according to the principles of Helsinki.

Patient demographics were retrieved from the hospital medical records. Most procedures were filmed with simultaneous recording of fluoroscopy, the operating field, PSA, and electrophysiology (EP) recording system, allowing us to reconstitute the procedure at specific timepoints.

With stylet-driven leads, iEGMs were recorded continuously during lead deployment by connecting the cathodal crocodile clip to the stylet, whereas the cable was disconnected during lumenless lead deployment, until the John Jiang rotational connector cable became available at our centre towards the end of the study.

ECG and iEGMs were recorded using a Boston Scientific Labsystem Pro EP recording system. Filter settings for the surface ECG were 0.05–150 Hz; for the unfiltered IEGM channel, they were 0.5–500 Hz, and for the filtered iEGM channel, 30–500 Hz. Gain settings were optimized for the measurements but were by default 4 × for the ECG, 32 × for the unfiltered iEGM and 128 × for the filtered iEGM channels. Measurements were performed using digital calipers at 100 mm/s sweep speed. Analysis of the filtered channel was also performed at 100 mm/s. All measurements were performed by H.T. and H.B.

For the purposes of this study, we used the same QRS waveform nomenclature as for the surface ECG, without distinguishing between small and large waveforms (e.g. q or Q) for the sake of simplicity, and as no cut-off values exist.

**Figure 1:**
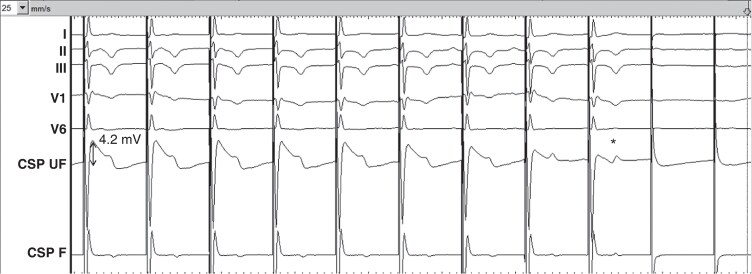
Macroperforation with complete loss of capture during continuous unipolar pacing at 5 V/0.5 ms. Note the fall in current of injury (COI), initially measured at 4.2 mV (double-sided arrow), falling to 0.8 mV just before loss of capture. Note that the COI falls to a lesser extent during repolarization (i.e. during the T-wave, annotated by *) than during depolarization (i.e. during the QRS complex, where the measurement was consistently taken in all patients). This patient was being implanted with a 3830 lead, with interruption of rotations, while the paced COI was approximately 12 mV. The perforation occurred spontaneously over the following 30 s (possibly due to release of torque build-up within the delivery catheter). Sweep speed is 25 mm/s. CSP UF = unfiltered conduction system pacing channel; CSP F = filtered conduction system pacing channel.

For consistency, COI amplitude was measured at the maximum value of the COI dome during myocardial depolarization (i.e. during the QRS complex or at the J-point and not during the T-wave; see *Figure 1*). As a decrease in COI amplitude over the minutes following fixation is usual, waveform measurements were performed within 1 min of lead deployment. In case of transitions to selective capture during decrementing output, waveforms were only analysed during non-selective capture in order to reflect the findings encountered with the default output of 5 V/0.5 ms delivered by continuous pacing during lead deployment.

Microperforation was defined as a sensed COI amplitude of ≤5 mV with a drop in unipolar lead impedance by >200 Ω from the peak value, with myocardial and/or conduction system capture at 5 V/0.5 ms unipolar pacing (which is the default pacing output of the PSA). Macroperforation was defined as loss of unipolar capture at a pacing output of 5 V/0.5 ms, with or without free mobilization of the lead in the left ventricular cavity. In case of perforation, leads were repositioned at a distant site (guided by fluoroscopy and pacemapping).

In order to avoid bias and autocorrelation, only a single set of values was captured for each patient (i.e. data from only one instance of perforation in patients with multiple perforations). In cases that had both micro- and macroperforation, only data for microperforation were recorded, as these were felt to be of greater interest to evaluate (as macroperforation is easy to diagnose).

Lead position was defined either as anterior (superior), septal, or posterior (inferior) based upon QRS axis of paced complexes being >^+^30°,^+^30° to ^−^30°, or <^−^30°, respectively, as previously described.^8^

### Statistical analysis

As the Kolmogorov–Smirnoff and Shapiro–Wilk tests showed non-Gaussian distribution of the iEGM data, the Wilcoxon and Mann–Whitney tests were used to evaluate differences in continuous variables between groups. In cases with absent waveforms (e.g. absence of a Q wave), cell values were reported as 0 unless specified otherwise. Fisher’s exact test was used for evaluating nominal data. Correlation was calculated using Pearson’s test, and agreement was evaluated using Bland–Altman analysis. GraphPad Prism 8.0 (GraphPad Software Inc.) was used for statistical analyses. A two-tailed *P* < 0.05 was considered statistically significant.

## Results

Of the 798 patients undergoing LBBAP implantation, 129 (16%) had perforation, of whom 73 (9%) only had microperforation, 42 (5%) only had macroperforation, and 14 (2%) had both micro- and macroperforation. Of these 129 patients with perforations, 92 (71%) patients (65 with microperforation and 27 with macroperforation) had analysable data retrieved from the EP recording system. Patient demographics are shown in *Table 1*.

**Table 1:** Baseline and procedural characteristics. N (%) or mean ± standard deviation

Patient demographics (*n* = 92)	
Age (years)	79 ± 11
Female	34 (37%)
**Medical history**	
Hypertension	59 (64%)
Diabetes	30 (33%)
Renal insufficiency	17 (22%)
Ischemic heart disease	36 (39%)
CABG	3 (3%)
Dilated cardiomyopathy	7 (8%)
Cardiac amyloidosis	3 (6%)
Valve surgery	7 (8%)
TAVI	8 (9%)
Ejection fraction, %	52 ± 15
**Indication**	
SSS/tachy-brady syndrome	6 (7%)
AV block	59 (64%)
AF + AV node ablation	6 (7%)
Heart failure/CRT	12 (13%)
Syncope	9 (10%)
**Baseline ECG**	
Sinus	70 (76%)
AF/flutter	22 (34%)
Intrinsic QRS duration (ms)	133 ± 34
Normal QRS morphology	35 (38%)
LBBB	18 (20%)
LAHB	3 (3%)
RBBB	10 (11%)
RBBB + hemiblock	11 (12%)
IVCD	4 (4%)
Ventricular intrinsic rhythm	1 (1%)
Ventricular paced rhythm	10 (11%)
** Implanted device**	
VVI/DDD pacemaker	78 (85%)
CRT-*P*	6 (7%)
CRT-D	8 (9%)
Lumenless lead	31 (35%)
Stylet-driven lead	57 (62%)
QRS transition or fascicular potential	30 (33%)

CABG, coronary artery bypass graft surgery; TAVR, Transcatheter aortic valve implantation; SSS, sick sinus syndrome; AF, atrial fibrillation; CRT, cardiac resynchronization therapy; LBBB, left bundle branch block; LAHB, left anterior hemiblock; RBBB, right bundle branch block; IVCD, intraventricular conduction delay.

The sites of pacing where septal perforations were encountered were anterior (superior), septal, and posterior (inferior) in 18%, 45%, and 37%, respectively, according to the paced QRS morphology and current criteria.^8^

### Waveforms during perforation compared to final lead position

**Figure 2:**
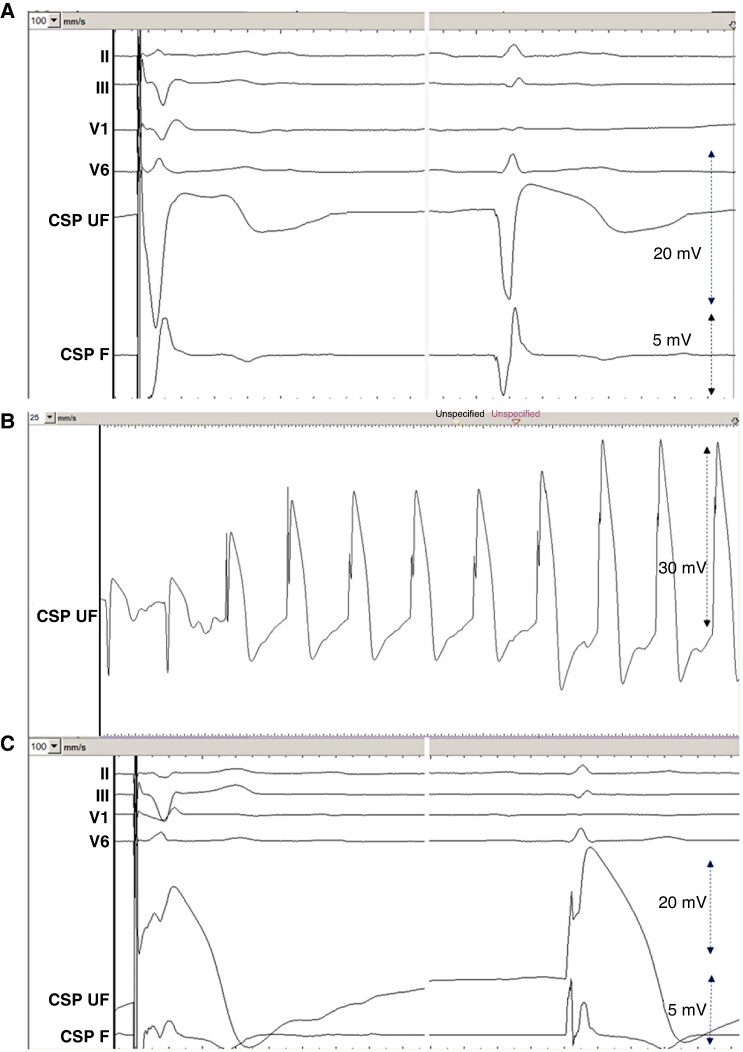
Typical waveforms in a patient with narrow QRS during (*A*) Microperforation with pacing and sensing. During sensing, note the deep and wide QS wave (62 ms, 12.2 mV) and low COI (3.1 mV) in the unfiltered channel and no high-frequency multiphasic component in the filtered channel. (*B*) Sensed waveform during gradual pullback of the perforated lead. Note the sudden change in electrogram waveform from a QS to an R pattern and thereafter a gradual increase in COI amplitude. (*C*) Final lead position with pacing and sensing. During sensing, note the tall R-wave (13.5 mV) and high COI amplitude (21.3 mV) with the high-frequency components in the filtered electrogram, timed at QRS onset (arrow). Sweep speed is shown in mm/s in the top left of each panel. CSP UF = unfiltered conduction system pacing channel. CSP F = filtered conduction system pacing channel.

**Figure 3:**
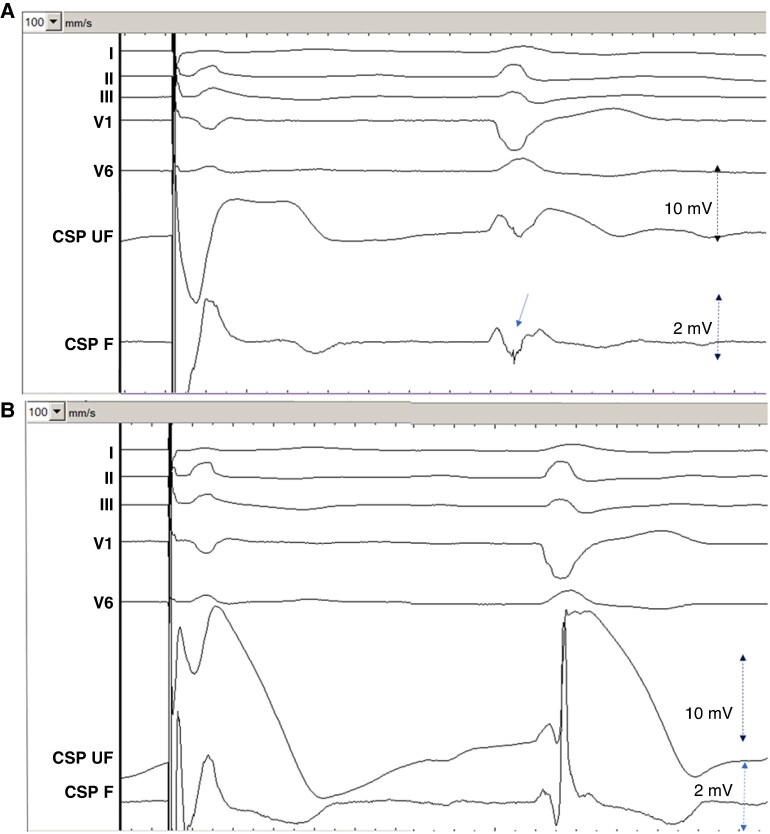
Patient with left bundle branch block. *(A*) Microperforation with electrogram during pacing (left) with a QS waveform and during sensing (right) with a broad RS waveform in the unfiltered channel. Note the sharp high-frequency component in the filtered channel, timed late after QRS onset (arrow). *(B*) Final lead position with electrogram during pacing and sensing. Sweep speed is 100 mm/s. CSP UF = unfiltered conduction system pacing channel (scale 32x); CSP F = filtered conduction system pacing channel (scale 128x).

**Figure 4:**
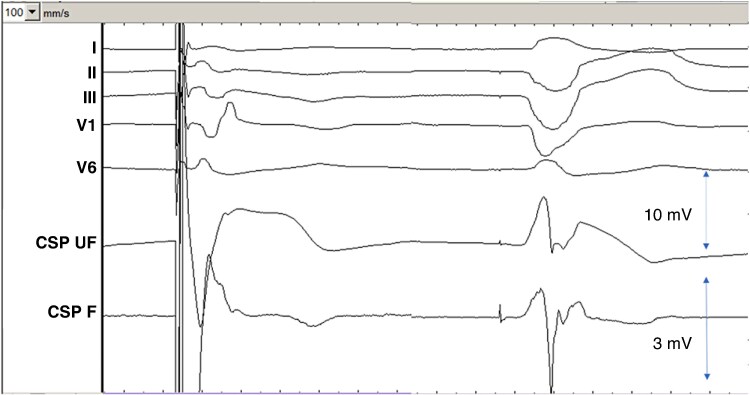
Microperforation during pacing (left) and during sensing (right) of right ventricular paced rhythm. Note the QS iEGM morphology during pacing and the broad R waveform during sensing of the backup paced rhythm. Sweep speed is 100 mm/s. CSP UF = unfiltered conduction system pacing channel (scale 32x); CSP F = filtered conduction system pacing channel (scale 128x).

**Figure 5:**
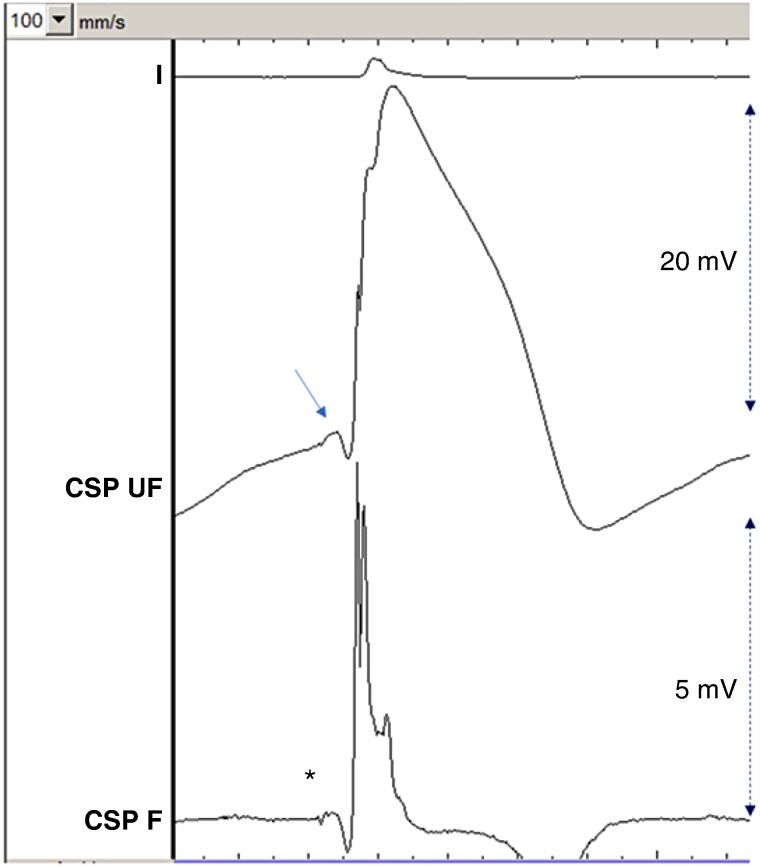
Example of lead in its final position with a small q-wave (QR morphology of the waveform), with a myocardial current of injury amplitude of 21 mV. Note the small fascicular potential in the filtered channel (asterisk) with a visible current of injury in the unfiltered channel (arrow). Multiphasic sharp high-frequency potentials are visible in the filtered channel at the onset of the QRS complex. Sweep speed is 100 mm/s. CSP UF = unfiltered conduction system pacing channel (scale 32x); CSP F = filtered conduction system pacing channel (scale 128x).

Data for the entire cohort during perforation and at the final lead position are shown in *Table 2*. The sensed iEGM waveform patterns are shown in *Table 3*. Typical waveforms are illustrated in *Figures 2–5*. During the course of the implantation, six patients without temporary pacing (two with narrow QRS/non-LBBB and four with LBBB) became pacemaker-dependent and did not have final sensed cycles. The COI amplitude was higher at the final lead position than during perforation, although three patients had COI <5 mV (minimum 2.1 mV), which were accepted by the implanting physician based upon clinical decision. Sharp, high-frequency multiphasic components of the ventriculogram were visible in the filtered iEGM channel in a minority (32%) of patients with perforation and in the majority (88%) of patients at final lead position (see *Table 2*).

**Table 2:** Data for the entire patient cohort of iEGM waveforms during perforation and at final lead position during pacing and sensing.

	Perforation			Final position		
iEGM parameter	*Paced* ^a^	*Sensed*	*P*-value	*Paced*	*Sensed* ^b^	*P*-value
Q present	53/62 (85%)^c^	50/92 (54%)	<0.0001	16/92 (17%)*	15/86 (17%)^#^	1.0
Q amplitude (mV)	5.6 [2.8–10.0]^c^	1.0 [0.0–6.9]	< 0.0001	0.0 [0.0–0.0]*	0.0 [0.0–0.0] ^#^	0.049
Q duration (ms)	72 [46–87]^c^	32 [0–66]	< 0.0001	0 [0–0]*	0 [0–0] ^#^	0.038
R present	NA^d^	48/92 (52%)	—	NA^d^	84/86 (98%)^#^	—
R amplitude (mV)	NA^d^	0.3 [0–1.8]	—	NA^d^	8.6 [3.5–12.1]^#^	—
R duration (ms)	NA^d^	34 [16–49]	—	NA^d^	29 [22–41]†	—
S present	0/62 (0%)	75/92 (82%)	<0.0001	0/92 (0%)	26/86 (30%)^#^	<0.0001
S amplitude (mV)	—	5.5 [2.2–8.5]	—	—	0.0 [0.0–1.8]	
S duration (ms)	—	56 [28–70]	—	—	0 [0–11]	
COI amplitude (mV)	4.1 [3.0–5.3]	3.0 [1.5–4.1]	< 0.0001	13.0 [9.7–17.4]*	14.0 [9.2–17.5]^#^	0.37
HF component	NA	29/92 (32%)	—	NA	76/86 (88%)^#^	—

In case of a QS morphology, the waveform counted towards both Q as well as S. Absence of a waveform (e.g. Q-wave) was counted as 0 value. Differences between the groups were calculated using the Wilcoxon and Fisher’s exact tests. Waveform durations and amplitudes displayed as median with interquartile range.

COI, current of injury; iEGM, intracardiac electrogram; HF, high-frequency component in filtered intracardiac electrogram; ms, milliseconds; mV, millivolts; NA, not available.

^a^Discounting 27 patients with macroperforation who had loss of capture and 3 patients in whom pacing had not been performed during perforation.

^b^Discounting six patients without intrinsic rhythm and without temporary pacing (who had become pacemaker-dependent during the course of the implantation).

^c^All QS waveforms.

^d^During pacing, R-waves were usually difficult to distinguish from the pacing artefact and/or COI, so were not measured.

* *P* < 0.0001 compared to paced during perforation.

^*^*P < 0.0001 compared to sensed during perforation.

**P = NS compared to sensed during perforation.

**Table 3:** Sensed intracardiac electrogram patterns for the entire cohort and with subgroups based on QRS morphology, during perforation (micro- and macro-) and at the final lead position.

	Perforation			Final position		
Sensed iEGM pattern	All patients (*n* = 92)	Narrow QRS/non-LBBB (*n* = 63)	LBBB/paced (*n* = 29)	All patients (*n* = 86^a^)	Narrow QRS/non-LBBB (*n* = 61)	LBBB/paced (*n* = 25)
QS	47%	67%	3%	3%	3%	—
QR	4%	2%	—	13%	16%	4%
RS	33%	25%	48%	22%	23%	24%
R	14%	2%	45%	56%	49%	72%
Multiphasic^b^	14%	5%	3%	6%	8%	—

iEGM, intracardiac electrogram; LBBB, left bundle branch block.

^a^Six patients without backup ventricular pacing had become pacemaker dependent during the course of the implantation and did not have final sensed beats.

^b^Includes QRS, RSR, QRSR, etc.

### Subgroup analysis according to baseline ECG

**Figure 6:**
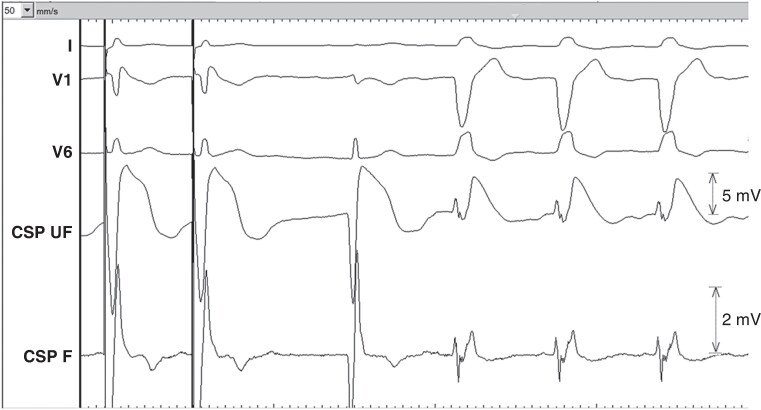
Post-pause QRS narrowing in a patient with underlying left bundle branch block with microperforation. Note the deep and wide QS waveform during sensing of the narrow cycle, with broad RS morphology during resumption of left bundle branch block. Sweep speed is 50 mm/s. CSP UF = unfiltered conduction system pacing channel; CSP F = filtered conduction system pacing channel.

It became apparent that patients with LBBB or temporary RVP display a distinct sensed iEGM pattern during perforation compared to those with a narrow QRS or non-LBBB conduction system disorder. A broad R/RS pattern was observed in 93% of these patients during perforation, whereas a broad QS pattern was the predominant finding in 67% of the patients with narrow QRS/non-LBBB (see *Table 3*). One patient with perforation and LBBB had post-pause QRS narrowing, with a QS iEGM waveform during the narrow cycle, reverting to a R/RS waveform with recurrence of LBBB (see *Figure 6*). We observed the same phenomenon in three other patients at our centre, who did not have perforation and therefore were not included in the study population. The COI amplitude was slightly higher during the narrow QRS cycle than during LBBB.

There were 13 patients with left anterior fascicular block (either isolated or associated with right bundle branch block). Only one patient showed a dominant R waveform during perforation, with the lead being implanted in the anterior (superior) region in this patient—all other patients had a dominant negative waveform with the leads implanted in the septal or posterior (inferior) regions.

The R-waves in patients with LBBB or RVP compared to those with a narrow QRS or non-LBBB conduction disorder during perforation were of higher amplitude: 2.6 [1.4–3.8] mV vs. 1.5 [0.7–2.8] mV, *P* = 0.028, and were significantly wider: 48 [32–70] ms vs. 25 [12–35] ms, *P* < 0.0001.

**Figure 7:**
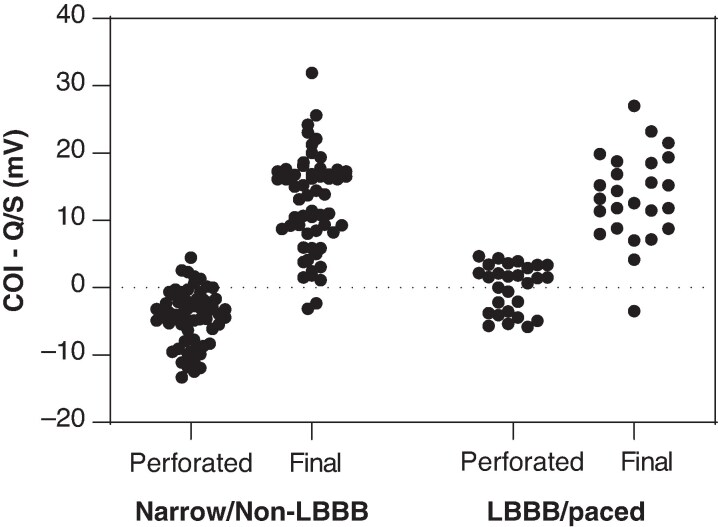
Plot of difference between current of injury amplitude (COI) and the negative component of the waveform (Q or S wave). Negative values indicate greater Q or S amplitude compared to COI. The datasets are separated according to baseline QRS (normal or non-LBBB vs. LBBB/paced), during perforation, and at final position.

We investigated whether perforation was associated with a greater amplitude of the negative waveforms (Q or S) with respect to the COI amplitude during *sensing*, to devise new criteria for evaluating perforation beyond COI amplitude as a stand-alone iEGM parameter. Data were analysed separately according to baseline QRS, due to the significant differences in iEGM waveforms; the results are shown in *Figure 7*. In patients with narrow QRS/non-LBBB, a sensed Q or S > COI amplitude was present in 54/63 (86%) during perforation and in 4/61 (7%) of patients at final lead position (COI amplitude was low at <5.5 mV in all of these four cases and <5 mV in two cases). In these patients, the criterion had a sensitivity of 86% with a specificity of 93% and an accuracy of 90% for diagnosing perforation. In patients with LBBB or RVP, a Q or S > COI amplitude was only present in 12/29 (41%) patients during perforation (it was less discriminative due to the high prevalence of an R-wave) and in 1/25 (4%) patients at the final lead position. At the final lead position, a deep and large QS waveform was only observed in one patient during sensing (COI was 13.7 mV, with amplitude of QS < COI).

The *paced* iEGM waveforms were similar in patients with a narrow QRS/non-LBBB compared to those with LBBB/RVP, both during perforation (deep and wide Q-wave) as well as at final lead position (distinct R or fused with COI).

We also observed that ventricular premature beats with an LBBB pattern had the same characteristic broad R or RS iEGM pattern as patients with LBBB or RVP, although this was not systematically studied.

High-frequency multiphasic potentials, when present, were typically at QRS onset in patients with narrow QRS/non-LBBB, whereas they were timed later in patients with LBBB/RVP (see *Figure 3A*).

### Subgroup analysis according to micro- and macroperforation

The sensed COI amplitude was significantly lower during macroperforation (*n* = 27) than during microperforation (*n* = 65): 1.6 [1.0–2.5] mV vs. 3.4 [2.2–4.2] mV, *P* = 0.001. There were no significant differences in the other waveform (Q, R, or S) amplitudes or durations between micro- and macroperforations.

In patients with a narrow QRS or non-LBBB, a QS waveform was present in 19/21 (90%) of patients with macroperforation and in 23/42 (55%) with microperforation (*P* = 0.005). In patients with LBBB or RVP, a QS morphology was encountered in 0/6 (0%) with macroperforation (all had R or RS waveforms) and in 1/23 (4%) with microperforation (*P* = 1.0). The only patient with LBBB morphology and a QS waveform had sharp multiphasic components of the ventriculogram in the filtered iEGM channel at the onset of the QRS complex (as was usually the case in patients with a normal or non-LBBB QRS morphology), suggesting that this patient may not have had true LBBB but rather more peripheral conduction disease with preserved septal activation.

A sharp multiphasic component of the ventriculogram of the filtered channel was observed in 26/65 (40%) patients with microperforation and in only 3/27 (11%) with macroperforation (*P* = 0.007).

### Waveforms during pacing vs. sensing

The COI amplitude with microperforation was poorly correlated during pacing and sensing (r = 0.42) with a bias of 1.2 mV (higher paced COI) and 95% limits of agreement of −2.5 to 5.0 mV. At the final lead position, the correlation was good at 0.92 with a bias of −0.4 mV and 95% limits of agreement of −4.8 and 4.0 mV. A deep and broad Q was present in the majority of cases during pacing and sensing with microperforation, whereas a small and narrow Q wave was present in 17% of patients at the final lead position in both sensed and paced beats (see *Table 2*).

### Electrical parameters and clinical follow-up

At implantation and after a median follow-up of 1.1 [0.1–1.7] years, the capture thresholds were 0.75 [0.6–0.9] V/0.4 ms and 0.75 [0.6–1.0] V/0.4 ms, respectively (*P* = 0.24). Impedances were 638 [559–711] Ω and 547 [476–586] Ω, respectively (*P* < 0.0001). Sensing amplitudes were 8.3 [7.0–11.1] mV and 11.8 [98.0–16.7] mV, respectively (*P* = 0.002).

None of the patients required lead revisions. A total of nine patients died, five of unknown causes, two of non-cardiovascular causes, one of haemorrhagic stroke, and one of sudden death. This was a 76-year-old male with 45% left ventricular ejection fraction whose pacemaker follow-up was normal on the day following implantation and who died suddenly 6 weeks later.

## Discussion

The main findings of our study were that (1) sensed waveforms (Q, R, and S wave duration and amplitude) are significantly different during perforation compared to the final lead position, in addition to COI amplitude; (2) patients with LBBB/RVP have a distinct broad R/RS sensed waveform during perforation compared to those with a narrow QRS/non-LBBB, who most often have a QS pattern; (3) a Q or S > COI amplitude is frequently encountered during perforation in patients who have a narrow QRS/non-LBBB; (4) a sharp multiphasic component of the ventriculogram in the filtered channel is less frequently seen during perforation compared to the final position, particularly with macroperforation; and (5) Waveforms may significantly differ during pacing and sensing with microperforation. These findings are discussed subsequently.

In sensed beats, during perforation compared to the final lead position, the negative components of the iEGM waveform (Q and/or S-waves) are of significantly greater amplitude and duration, whereas the positive component (R-wave) is of lower amplitude. This can be explained by the fact that with perforation, the wavefront is predominantly directed away from the lead tip (particularly in patients with normal septal activation). However, due to the very rapid change in waveform morphology over a short distance (i.e. with relatively little change in overlying myocardial thickness, see *Figure 2B*), additional factors are likely to contribute to the findings. The fall in COI amplitude also means that the negative forces are countered to a lesser extent, resulting in deepening and broadening of the Q or S waves in the unfiltered channel, which records low-frequency far-field signals. The contribution of this factor may be elucidated by studying the evolution of waveform morphology with the natural fall in COI over time, which we did not evaluate. It is important to note that low-amplitude and narrow Q-waves may be observed at the final lead position, even at locations with a high COI (see *Figure 5*). This may be explained by cases with a thick layer of connective tissue (measuring up to 0.6 mm), which surrounds the left bundle branch.^22^ The initial activation wavefront may be directed away from the lead tip during sensing if the helix is embedded in inert tissue. It is important to note that the fall in R-wave amplitude with perforation may be automatically measured by the filtered channel of a PSA, but this does not equate to the COI amplitude, which may differ significantly, as we have previously shown.^20^

**Figure 8:**
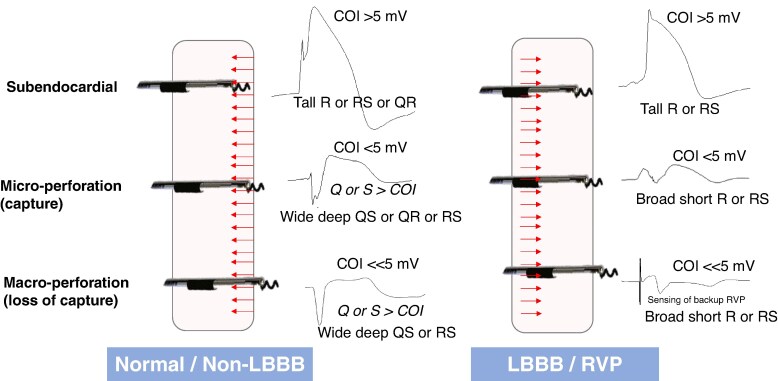
Intracardiac unipolar LBBAP unfiltered sensed electrogram waveforms at different lead depths and with different QRS morphologies. In patients with a normal QRS/non-LBBB, the interventricular septum is activated from left to right. With a sub-endocardial LBBAP lead, the wavefront is initially directed towards the lead tip, resulting in an R wave. With micro- and macro-perforation, the wavefront is directed away from the lead tip, resulting in a predominantly negative and broad (Q or S) waveform, possibly also due to the fall in COI amplitude, which has a lesser counteracting effect. In patients with LBBB and RVP, septal activation is from right to left, resulting in a predominantly positive R/RS waveform at all lead depths. COI = current of injury; LBBAP = left bundle branch area pacing; LBBB = left bundle branch block; RVP = right ventricular pacing.

Previous studies in smaller patient cohorts^11,17^ and in experimental swine models^17,19^ have reported QS iEGM waveforms with perforation. For example, Kato et al.^17^ reported that a QS waveform was observed intra-operatively in 9/11 (82%) patients with partial perforation suggested by post-operative echocardiography, vs. 0/41 (0%) patients without partial perforation. In our larger series of 92 patients with perforation, a QS waveform was mainly limited to patients with a narrow QRS and non-LBBB conduction disorders. The 29 patients with LBBB or backup RVP mostly had a distinctive broad R or RS sensed waveform with perforation. Implanting physicians should therefore not rely on the absence of a Q-wave to rule out perforation in these patients. Our hypothesis is that the wavefront during left-to-right septal activation (as is the case in narrow QRS and non-LBBB) is predominantly directed *away* from the lead tip with perforation, resulting in a dominant negative (Q and/or S) waveform. Conversely, in LBBB and RVP, the wavefront *approaches* the lead tip from the right septum, resulting in a dominant R-wave, which is broad due to its far-field component (see *Figure 8*). The finding may also be observed in the case of fascicular block and concordant lead position, for example, left anterior fascicular block and anterior (superior) lead placement. Another factor which is likely to impact the iEGM waveform is the apex-to-base or base-to-apex activation. The cases with change in iEGM morphology during post-pause QRS narrowing of LBBB (see *Figure 6*), as well as the observation of dominant R-waves with LBBB-type ventricular premature beats, underline the impact of wavefront activation on the iEGM waveform.

We observed that Q or S > COI amplitude was a marker of perforation in patients with narrow QRS/non-LBBB. This parameter, which is easy to eyeball during implantation, is described for the first time and may be useful for determining if perforation is present in the case of borderline COI amplitude (around 5 mV).

The filtered iEGM channel is usually only used for visualizing conduction system potentials. We found that sharp multiphasic components of the ventriculogram are more often present at the final lead position than during perforation, and are particularly rare in case of macroperforation. These high-frequency signals likely reflect local near-field myocardial activation and may be a marker of myocardial tissue contact of the helix. This is also supported by observations regarding their timing, that is, early with respect to the QRS in case of narrow QRS/non-LBBB, and late in case of LBBB/RVP.

During pacing, Q-waves were observed in the majority (85%) of cases during perforation and in a minority (17%) of cases at the final lead position. During non-selective LBBAP, the initial wavefront is expected to always be directed away from the lead tip due to centrifugal activation. However, the COI may counteract Q-wave amplitude and duration. As the COI drops, one observes a deepening and broadening of the Q-wave. During pacing with non-selective LBBAP, S-waves were never observed, contrary to during sensing. We observed cases with RS-waves with pacing during selective capture, but this was not systematically evaluated.

Delivering continuous pacing during lead deployment is useful to monitor not only the paced QRS morphology, but also a fall in COI amplitude and the appearance of a broad and deep Q-wave, which may herald perforation. However, the sensed iEGM provides richer information (such as the presence of fascicular potentials and validated cut-offs of COI for diagnosing perforation). As previously reported,^20^ paced and sensed COI may differ by up to 5 mV. Pacing should therefore be intermittently interrupted to analyse the sensed waveforms when the paced COI amplitude reaches approximately 10 mV.^20^ As the sensed COI falls below 10 mV, further lead rotations should be performed cautiously, and the iEGM parameters listed in the *Graphical Abstract* should be carefully monitored to avoid overshooting the target zone and end up with perforation.

There is consensus that in case of macroperforation, the lead should be newly repositioned rather than simply withdrawn to the target position.^8^ However, in case of suspected microperforation, it is unclear whether the lead needs to be repositioned or whether it may be simply left in place. A recent study in 95 patients showed that partial perforation, as diagnosed by post-operative transthoracic echocardiography, was not associated with adverse outcomes during 24-month follow-up.^17^ However, transthoracic echocardiography may falsely indicate partial perforation due to side-lobe artefacts,^23^ and tenting of the left ventricular endocardium may not always correspond to true microperforation. Three of our patients had suspected microperforation at their final lead position, which were nevertheless accepted by the operator, without adverse sequelae. However, one of our patients (not included in the present cohort as he did not fulfill the criteria of COI < 5 mV within 1 min of lead deployment) had suspected spontaneous microperforation, which had evolved progressively over the 13 min following lead positioning, with a final COI amplitude of 4.1 mV and a deep, broad S-wave. The lead was left in position due to excellent thresholds and the continued visualization of a fascicular potential, but macroperforation with loss of capture was diagnosed on the day following implantation and confirmed upon lead revision. Therefore, although complications may be rare, it seems wise to reposition leads with suspected microperforation to avoid progression to macroperforation or to lead dislodgement. Prospective evaluation of leads with suspected microperforation that were left in place, or retrospective waveforms analysis of patients with late perforations, may clarify this dilemma.

### Study limitations

The main limitation of our study is that microperforation was not confirmed with peroperative imaging (e.g. intracardiac echocardiography). However, the COI amplitude cut-off of <5 mV to diagnose perforation has previously been validated in the animal model using the same filters as in our study, albeit in a limited dataset (11 perforations in 3 swine).^19^ In order to increase specificity, we also required a drop in pacing impedance of >200 Ω, which has been proposed as a parameter to monitor perforation in the European Heart Rhythm Associaion consensus document on CSP implantation (although this cut-off was based upon operator experience rather than scientific data).^21^

Our definition of micro- and macroperforation differs from previous reports, which used imaging.^17,19^ However, pacing impedance, COI amplitude, and loss of capture (along with the waveform parameters which we newly describe) are practical and pragmatic to diagnose perforation, as per-operative imaging is usually not feasible. Contrary to our definition of macroperforation, myocardial capture may be successful at thresholds <5 V/0.4 ms with complete perforation of the lead helix if there is contact with the adjacent papillary muscle. However, we believe this to be a rare entity and notable sudden changes in QRS morphology are to be expected in these instances. As iEGM waveforms are impacted by filters,^20^ our results may not be applicable to different settings. Sensed waveforms were only evaluated during RVP and may have differed with backup pacing from different sites (e.g. from the coronary sinus). A number of cases at the final lead position may have been microperforations (three patients had a COI < 5 mV and others were borderline just above 5 mV); this may have confounded the results. However, a lesser difference between groups would lead to more conservative results. The evaluation of high-frequency components in the filtered iEGM was subjective and therefore prone to inter-observer variability, which was not tested. Furthermore, it is unknown if this parameter may have performed differently using other high-pass filter settings.

## Conclusion

Conduction system pacing is a paradigm shift in device implantation, whereby intracardiac signals play a major role in guiding the procedure, much as with electrophysiological interventions. There is a wealth of information that may be gleaned by carefully analysing iEGMs during LBBAP implantation. Our study provides a quiver of new parameters beyond just COI amplitude for evaluating the presence of LBBAP lead perforation. The ability to better forecast impending perforation should enable more effective and safer procedures, for the benefit of our patients.

## Data Availability

The data underlying this article will be shared on reasonable request to the corresponding author.
